# A genome-wide IR-induced RAD51 foci RNAi screen identifies CDC73 involved in chromatin remodeling for DNA repair

**DOI:** 10.1038/celldisc.2015.34

**Published:** 2015-12-01

**Authors:** Patrick Herr, Cecilia Lundin, Bastiaan Evers, Daniel Ebner, Christina Bauerschmidt, Guy Kingham, Timea Palmai-Pallag, Oliver Mortusewicz, Oliver Frings, Erik Sonnhammer, Thomas Helleday

**Affiliations:** 1 Science for Life Laboratory, Division of Translational Medicine and Chemical Biology, Department of Medical Biochemistry and Biophysics, Karolinska Institutet, Stockholm, Sweden; 2 Target Discovery Institute, Nuffield Department of Medicine, University of Oxford, Headington, UK; 3 CR-UK/MRC Oxford Institute for Radiation Oncology, Department of Oncology, University of Oxford, Oxford, UK; 4 School of Life Sciences, University of Lincoln, Brayford Pool, Lincoln, UK; 5 Science for Life Laboratory, Bioinformatics Centre Stockholm, Department of Biochemistry and Biophysics, Stockholm University, Stockholm, Sweden

**Keywords:** genome-wide siRNA screen, RAD51, homologous recombination, tumor suppressor, CDC73, Parafibromin, DNA repair, chromatin remodeling

## Abstract

To identify new regulators of homologous recombination repair, we carried out a genome-wide short-interfering RNA screen combined with ionizing irradiation using RAD51 foci formation as readout. All candidates were confirmed by independent short-interfering RNAs and validated in secondary assays like recombination repair activity and RPA foci formation. Network analysis of the top modifiers identified gene clusters involved in recombination repair as well as components of the ribosome, the proteasome and the spliceosome, which are known to be required for effective DNA repair. We identified and characterized the RNA polymerase II-associated protein CDC73/Parafibromin as a new player in recombination repair and show that it is critical for genomic stability. CDC73 interacts with components of the SCF/Cullin and INO80/NuA4 chromatin-remodeling complexes to promote Histone ubiquitination. Our findings indicate that CDC73 is involved in local chromatin decondensation at sites of DNA damage to promote DNA repair. This function of CDC73 is related to but independent of its role in transcriptional elongation.

## Introduction

The DNA damage response is a safeguarding mechanism that ensures maintenance of the genomic integrity of cells. Aberrant DNA repair leads to genomic instability and cancer [1]. Two main pathways have been identified to repair DNA double-strand breaks (DSBs) in the cell. Non-homologous end-joining resolves DSBs by direct ligation of DNA ends and is therefore more error prone. Homologous recombination repair (HRR) requires an intact sister chromatid as a template [1] enabling error-free repair of DSBs. To find new regulators involved in HRR, we performed a genome-wide short-interfering RNA (siRNA) screen combined with ionizing radiation (IR). The cellular phenotype we scored by high content imaging was the absence of RAD51 recruitment to sites of DNA damage. RAD51 is a protein specifically involved in homology searching and strand pairing [2] and thus is essential in HRR-mediated DSB repair. We thereby aimed to enrich our candidate gene set for HRR-specific factors.

RNA-metabolizing enzymes have previously been identified to be involved in DNA damage response and HRR [3, 4]. Among many known HRR proteins, the top hit of our validation experiments was CDC73, which is encoded by the *HRPT2* tumor suppressor gene. CDC73 was shown to be associated with the PAF1/RNA polymerase II transcriptional elongation complex [5].

The chromatin state is pivotal for the ability of transcription factors and polymerases to access DNA. Histone modifications such as ubiquitination and methylation have been described to be required for temporary Histone eviction to allow accessibility of the DNA. Monoubiquitination of Lysine 120 on Histone 2B results in temporary displacement of H2B [6] and the ubiquitin ligases RNF20/40 bind CDC73 to mediate H2B monoubiquitination [7]. ATM was shown to directly activate RNF20/40 to mediate monoubiquitination at Lysin 120 in Histone 2B (H2BK120Ub). This direct activation in response to DNA damage is required for fast initiation of repair of DNA lesions [8, 9]. A direct role for RNA polymerase II in the DNA damage response was established through the detection and repair of DNA mismatches [10]. DNA double strand breaks in transcribed regions were shown to be preferentially repaired by HR to ensure precise repair of the lesion [11]. Monoubiquitination of H2B, however, was until recently only linked to ongoing transcription [12] and the mechanisms underlying damage-dependent Histone modification are just beginning to be revealed.

CDC73, as part of the PAF1 complex (PAF1c), is known to be mutated in a variety of cancers like parathyroid carcinoma, breast carcinoma and colorectal carcinoma [13]. Here we describe a novel role for CDC73 in the DNA damage response, which is independent of its transcriptional role with the PAF1c. We found CDC73 to interact with core Histones H2B and H3 and interestingly did we also discover interactions between CDC73 and members of the SCF/Cullin and INO80 chromatin-remodeling complexes like UBA1, CAND1, CUL1, FBXO21 and RUVBL2. These interactions are likely to be required to ensure efficient chromatin remodeling around the DSB to promote accessibility of the DNA for downstream repair events. In live-cell imaging experiments we show that CDC73 is required for efficient displacement of H2B from chromatin. Similar to loss of CDC73 does depletion of SCF/Cullin and INO80 complex components reduce the abundance of H2BK120Ub. We suggest a model in which CDC73 as part of the transcription complex recruits chromatin-remodeling factors of the SCF/Cullin and INO80 complexes and is required to modify the chromatin surrounding the DSB. Our study presents a new mechanism of action of CDC73, which provides important new insights into the tumor suppressor role of CDC73 in cancer.

## Results

### Genome-wide siRNA screen for regulators of HRR

**Figure 1:**
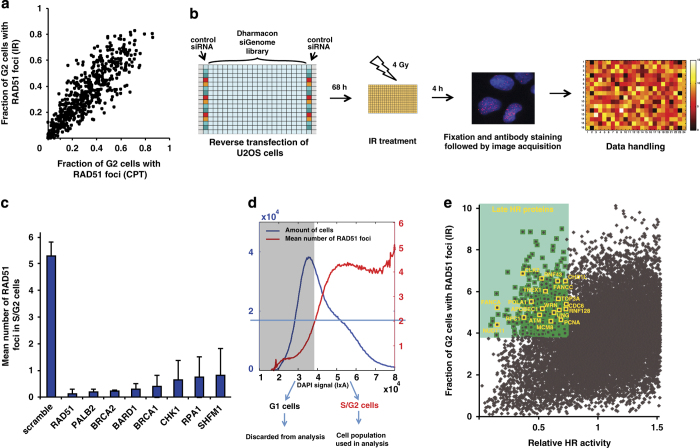
(**a**) Irradiation (4 Gy) and treatment with CPT (1 μM) show very close correlation in RAD51 foci formation. (**b**) Layout and results of the primary genome-wide siRNA screen. Cells were reverse transfected with a siRNA library and irradiated with 4 Gy 68 h after siRNA transfection. At 72 h after siRNA transfection, cells were fixed and imaged with an InCell Analyzer. Final data analysis was performed using MatLab (MathWorks, Natick, MA, USA). (**c**) Mean RAD51 foci numbers and s.d. for known HR proteins from the two duplicate runs. (**d**) Cells were sorted according to DNA content by only counting cells in the S/G2 phase. Plot showing mean number of RAD51 foci over a histogram for DAPI intensity. G1 to S-phase transition was set at DAPI intensity where mean number of RAD51 foci is 2 (horizontal blue line). (**e**) Illustration of late HR proteins in a green shading, which is defined by defective repair of the DR–GFP reporter but rather normal recruitment of RAD51.

HRR is induced by two-ended DSBs (caused by restriction enzymes or IR) or by one-ended DSBs formed at collapsed replication forks, caused, for instance, by the topoisomerase I–inhibitor Camptothecin (CPT) [14], and different enzymes may be involved in the subsequent HRR. Previously, a genome-wide screen using the I-SceI restriction endonuclease to induce HRR between GFP genes in the DR–GFP assay was performed [4]. Here, we study the formation of RAD51 foci as readout and compare one-ended and two-ended DSBs. Prior to the genome-wide screen, we carried out a smaller siRNA screen on 600 genes involved in DNA damage response and repair and scored for formation of RAD51 foci after two-ended (using IR) or one-ended (using CPT) DSBs to trigger HRR. To our surprise, we found a high correlation between the genes involved in forming RAD51 foci following CPT and IR (Figure 1a), indicating either that the same proteins are involved in two-ended and one-ended HRR or alternatively that a similar substrate for HRR is formed after both IR- and CPT-induced lesions. We previously demonstrated that IR-induced HRR is predominately triggered by secondary one-ended DSBs formed when replication forks collide into IR-induced lesions [15]. As IR- and CPT-induced HRR are predominately formed by the same one-ended DSBs, we decided to carry out a full genome-wide screen using IR.

For the genome-wide siRNA screen, we used siRNA pools targeting ~18 000 genes. Following siRNA knockdown, cells were irradiated, fixed after 4 h and stained for RAD51 and DNA content (Figure 1b). The screen was run in duplicates with a good correlation between the runs (*R*=0.68) (Supplementary Figure S1A). Negative (scrambled) and positive (RAD51) control siRNAs showed a significant difference in RAD51 recruitment (Supplementary Figure S1B). Furthermore, knockdown of known HRR proteins like *BRCA1, BRCA2* and *SHFM1* were found to significantly reduce levels of RAD51 foci formation, indicating a successful screen (Figure 1c, Supplementary Figure S1C).

HRR is known to be active only in the S/G2 phase of the cell cycle and we therefore excluded cells in the G1 phase from our analysis. Cells in G1 have no RAD51 foci but as they progress through S-phase the number of RAD51 foci increases (Figure 1d). To only analyze cells in the S/G2 phase, we set our G1 to S-phase cut-off at the DAPI (4′,6-diamidino-2-phenylindole) intensity where mean number of RAD51 foci equals 2. Cells in the G1 population were discarded and only data from cells in S/G2 phase were analyzed further. To account for plate-to-plate variation, as well as row and column effects, data were normalized using a three-dimensional (3D)-*B* score calculation to minimize systematic errors (Supplementary Figure S2A–D). As for *z*-score calculations, the 3D-*B* score value will be positive or negative depending on raw data being above or below population mean. In our screen, a low 3D-*B* score represents siRNAs decreasing RAD51 foci formation after irradiation. For the whole screen, the values for 3D-*B* score fell between −4.3 to +4.4 and almost 600 siRNAs had a 3D-*B* score lower than −2, that is, 2 s.d. from the mean of the whole screen (Supplementary Figure S1D, Supplementary Table S1).

### Hit detection and validation

We compared the results from our screen to the data set from another genome-wide HRR siRNA screen using I-SceI-induced two-ended DSBs and recombination between non-functional GFP genes as readout [4]. Interestingly, we found overall good correlation between the screens (Supplementary Figure S1E), indicating that proteins involved in HRR of two-ended DSBs are likely also involved in HRR of IR-induced lesions. To identify the proteins that suppress both HRR and RAD51 foci formation most effectively, we calculated a HRR score as the product from score values of both screens for each gene. Besides known HRR genes, this revealed new potential HRR suppressors (Supplementary Figure S1F). Proteins involved in catalyzing late recombination events are intact in forming RAD51 foci, which persist for a longer time. Hence, the gene products involved late in HR are likely to exhibit high RAD51 foci levels while poorly catalyzing HRR. Interestingly, we find proteins involved in replication (PCNA, RFC1, POLA1) and Holiday Junction resolution (TOP3A, WRN) producing high levels of RAD51 foci and low HRR activity, in line with these proteins being involved in late HRR steps (Figure 1e).

**Figure 2:**
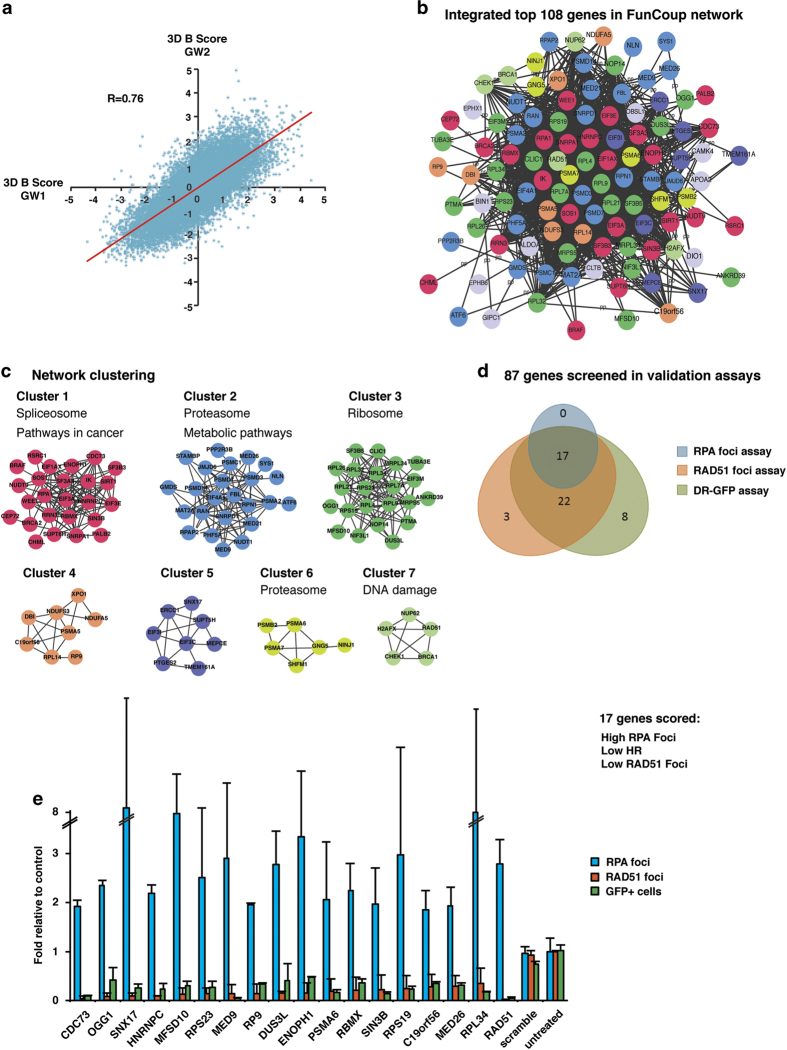
Schematic for how siRNAs were picked for validation. (**a**) 3D-*B* scores from the duplicate siRNA screens for RAD51 foci were combined with good correlation (*R*=0.76). (**b**) The top 200 candidate genes were mapped to the human FunCoup network resulting in a subnetwork of 108 genes. (**c**) Network clustering resulted in 7 sub-clusters from which 87 genes were selected for validation assays. The clusters provide more detailed functional annotation, determined by significantly enriched KEGG pathways. (**d**) Forty-two of the siRNAs that were validated showed significantly lower levels of RAD51 foci compared with control. Of these, 39 also had significantly lower HR-activity in the DR–GFP assay and 17 confirmed an HR-defect in all 3 validation assays (that is, low RAD51 foci levels, increased RPA foci levels and low levels of GFP+ cells in the DR–GFP assay). (**e**) Results of the 17 siRNAs that had significantly different levels compared with the control in the validation assays. Data showing mean fold values and s.d. from two independent experiments.

RNAi screening typically suffers from off-target effects and in particular off-target depletion of RAD51 protein has commonly been observed, resulting in false-positives [4]. Since our readout is RAD51 foci formation after DNA damage induction, our results would be highly affected if RAD51 were depleted in an off-target manner. Therefore, we performed network analysis on our data, assuming that if a gene is part of a RAD51 network and connected to another candidate gene, it is less likely that the effect is due to an off-target activity of the siRNA. First, data from the duplicates were merged by producing a rank list based on 3D-*B* score for each duplicate screen run and then ranks were summed to get a merged rank list. The number of genes to further analyze was chosen based on their correlation between the duplicate runs. Starting at 50 genes and increasing by 25 for each calculation, we found that a sample size of 200 genes gave the best correlation (Supplementary Figure S3), and therefore the top 200 of the merged rank list were chosen for further functional characterization using pathway analysis (Figure 2a). To characterize the genes in a network context they were mapped to the human FunCoup network (Figure 2b) [16, 17]. The resulting sub-network of 108 genes was further subjected to network cluster analysis using the MGclus method [18]. Using the hypergeometric probability distribution, we found a set of pathways significantly associated with the 200 genes, notably proteasome, ribosome, HRR and spliceosome (Figure 2c; Supplementary Table S2B). The clustering yielded a list of 7 clusters covering 98 genes, which were annotated using KEGG pathway annotations. This largely agreed with the all-gene analysis, but also revealed which cluster was specifically associated with particular pathways (Figure 2c; Supplementary Table S2A). Also, some new pathway associations appeared, such as ‘pathways in cancer’. Interestingly, the percentage of gene overlap with previous HRR screens is higher for 108 network connected genes and suggests that filtering our data for network connections enriches for true-positives and reduces the number of false-positives (Supplementary Tables S3A and S3B).

For experimental validation, we chose 87 candidate genes and used siRNAs from a different vendor than in the primary screen. Selected siRNAs were reverse transfected into U2OS cells using the same schematics as for the primary screen. First, we scored the reproducibility of the decrease in RAD51 foci formation after irradiation. Forty-two of the siRNAs showed a statistically significant decrease in RAD51 foci formation after IR (Figure 2d; Supplementary Table S4). For further validation of a HRR defect, we evaluated RPA foci formation after IR, as well as HRR activity in the DR–GFP assay. SiRNAs for 47 genes resulted in significant decrease in the number of GFP-positive cells in the HRR assay compared with the control population (Figure 2d; Supplementary Table S4). Thirty-nine of those siRNAs were the same as had scored positive in the RAD51 foci staining (Figure 2d). Finally, we scored RPA foci formation and found that 17 genes had statistically significant higher level of RPA foci and all of them also showed an HRR defect in the RAD51 foci and DR–GFP assays (Figure 2e, Supplementary Table S5). Depleting cells of RAD51 by siRNA lead to RPA foci accumulation (Supplementary Figure S4A) and a strong decrease in GFP^+^ cells (Supplementary Figure S4B). The top candidate gene from our confirmation experiments was CDC73 and we therefore decided to analyze its function in HRR in more detail.

### CDC73 loss generates genomic instability

**Figure 3:**
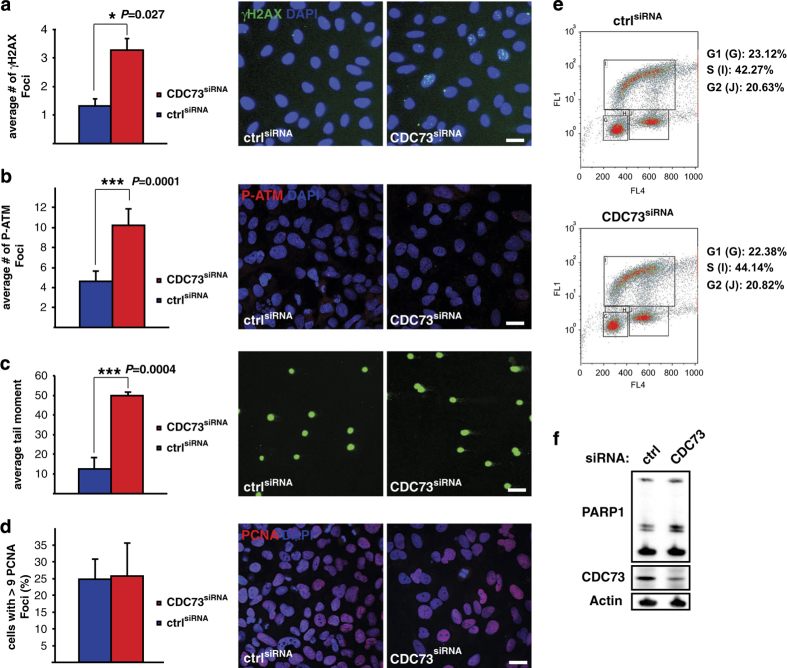
CDC73 silencing leads to genome instability. (**a** and **b**) Average number of γH2AX and P-ATM foci in U2OS cells depleted for CDC73 is significantly increased compared with control (*P=0.027 and ***P=0.001, respectively). (**c**) Comet Assay with siRNA-transfected U2OS cells. CDC73 depletion leads to significantly increased DNA damage as analyzed by the average tail moment (***P=0.004). (**d**) Normal S-phase distribution in CDC73-depleted cells based on PCNA staining. (**e**) CDC73 knockdown cells do not undergo cell cycle arrest and show a normal rate of BrdU incorporation as analyzed by FACS and propidium iodine counterstain. (**f**) CDC73 silencing does not lead to apoptosis measured by the amount of cleaved PARP1 on western blot. For all experiments *n*⩾2. Average and s.d. are plotted. *P*-values are calculated with Student’s *t*-test and scale bars represent 50 μm.

The top hit from our rescreen CDC73 is encoded by the *HRPT2* tumor suppressor gene and has been described as a component of the human PAF1/RNA polymerase II complex [5]. CDC73 is mutated in various types of cancer and absence of staining serves as a diagnostic marker [13]. The mode of action of CDC73 in cancer formation and progression, however, remains largely unknown. As we identified CDC73 as a potential regulator of HRR, we set out to determine the role of CDC73 in the maintenance of genomic integrity. Phosphorylation of ATM (P-ATM) and H2AX (γH2AX), both hallmarks of an activated DNA damage response induced on formation of DSBs, was significantly increased after knockdown of CDC73 (Figure 3a and b). Measuring the tail moment after alkaline single cell electrophoresis (Comet Assay) revealed increased fractionation of DNA in CDC73-depleted cells (Figure 3c), also indicating DSB formation. To rule out an effect on cell cycle progression of CDC73-depleted cells, we stained cells for PCNA recruitment (Figure 3d) and analyzed incorporation of BrdU (5-bromo-2′-deoxyuridine) in combination with propidium iodine by Fluorescence-activated cell sorting (FACS) (Figure 3e). In agreement with the data from our screen, we did not detect an effect on cell cycle progression, nor did we detect increased cleavage of PARP1 as a measure of cell death (Figure 3f). Taken together, depletion of CDC73 leads to increased DSBs and the activation of the DNA damage response.

Previous reports stated effects on cell cycle progression and cell viability upon depletion of CDC73 [19, 20]. The difference to what we observed can be explained by different experimental procedures. As CDC73 is involved in transcriptional elongation, we designed a short-term siRNA-silencing protocol (48 h) at which the CDC73 protein has largely disappeared, as measured by western blot, but secondary effects caused by affecting global transcription are not yet relevant. The use of five different siRNA sequences allowed us to safely conclude that our results are neither linked to an off-target effect on RAD51 directly nor to depletion of the RAD51 protein due to reduced transcriptional rates (Supplementary Figure S1G).

### Loss of CDC73 results in delayed repair of DSBs

**Figure 4:**
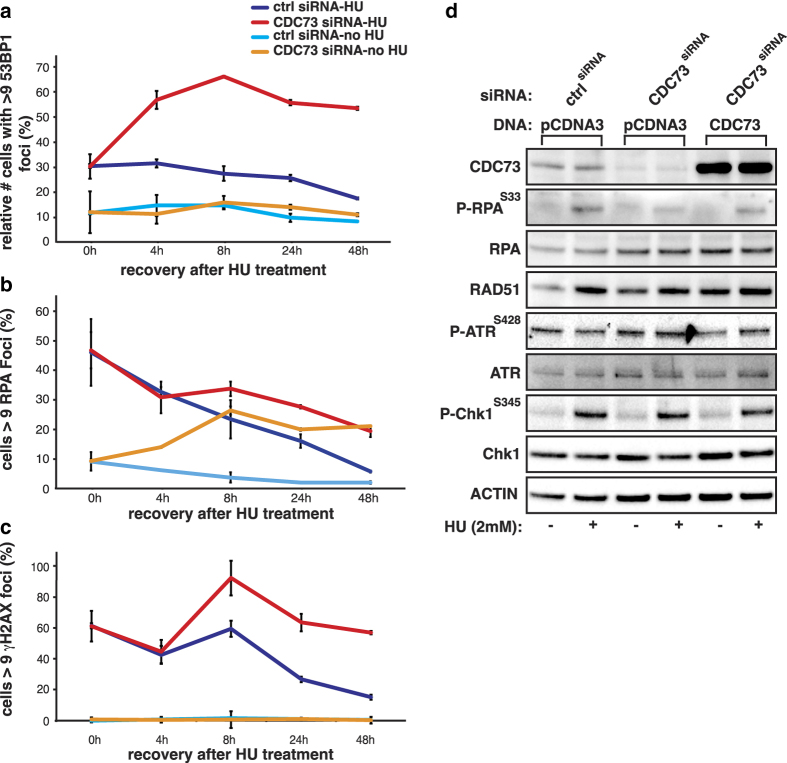
Loss of CDC73 results in delayed resolution of DNA DSB. Cells were followed over 48 h after being released from HU (2 mm per 12 h) treatment. (**a**–**c**) 53BP1, RPA and γH2AX are not released from sites of damage in CDC73 knockdown cells. (**b**) CDC73 knockdown alone steadily increases the accumulation of RPA. (**d**) RPA phosphorylation is reduced in CDC73 knockdown but ATR expression and activity are not affected (*n*⩾2). Average and s.d. are plotted. Scale bars represent 50 μm.

To study the dynamics of DNA repair in absence of CDC73, we treated U2OS cells with hydroxyurea (HU), which depletes the endogenous pool of dNTPs, subsequently leading to replication stress, stalled replication forks and DSBs [21]. Cells were released after 18-h treatment, and recruitment of γH2AX, 53BP1 and RPA was quantified at several time points over 48 h. Foci of all three markers were not resolved after depletion of CDC73 (Figure 4a–c). Interestingly, untreated CDC73-deficient cells also showed an accumulation of RPA foci over time (Figure 4b). These findings indicate a failure in resolving DNA damage at the level of or downstream of 5′ end resection or alternatively there could be more collision events between transcription and replication and more generation of unprotected single-stranded DNA. Indicative of a role for CDC73 in DNA repair at the level of efficient resection, we did observe a reduction of RPA S33 phosphorylation in HU-treated CDC73-silenced cells (Figure 4d). ATR-mediated RPA phosphorylation serves as a marker for repair factor recruitment [22]. We did not detect any changes in ATR S428 phosphorylation or on another ATR target Chk1 S345, arguing against an effect on the activation of ATR directly (Figure 4d). Alternatively the altered chromatin structure at DNA damage sites in CDC73-depleted cells could hamper phosphorylation of RPA by ATR.

### CDC73 is required for efficient H2B monoubiquitination and eviction

**Figure 5:**
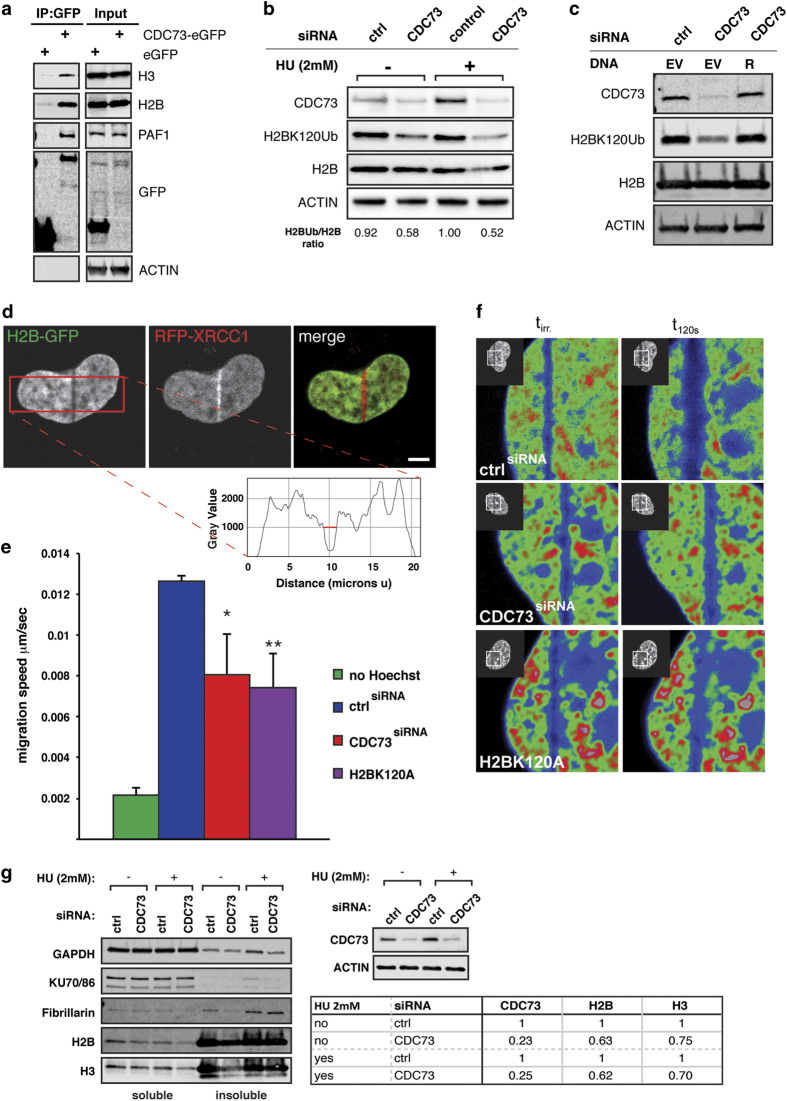
CDC73 silencing leads to impaired H2B ubiquitination and reduced DNA damage-induced H2B release. (**a**) CDC73 binds to Histones H2B and H3 in co-immunoprecipitation experiments. PAF1 serves as a positive control. (**b**) H2BK120Ub is reduced in CDC73 knockdown cells. The effect is enhanced after exposure to HU (2 mm). Calculated ratios (H2Bub/H2B) are indicated below the blot. (**c**) CDC73 silencing leads to decreased ubiquitination on H2BK120, which is rescued by expression of a siRNA-resistant CDC73 construct (R) (EV, empty vector control transfection). (**d**) Low energy laser irradiation (405nm) leads to DNA damage-induced recruitment of red fluorescent protein–XRCC1. (**e**) Cells were analyzed for the migration speed of H2B–GFP away from the site of DNA damage. Quantification was done using the plot profile tool in ImageJ (inlet) in the indicated area (red square in **d**) and the distance was measured at a defined gray value for start and end time points (red line). Each experiment was repeated at least 3 times and at least 10 cells were analyzed per experiment (Average and s.d. *P*-values are calculated with Student’s *t*-test and scale bar represents 5 μm). CDC73 silencing reduces the speed of H2B migration significantly (*P=0.017) and to a similar speed as the H2BK120A–GFP mutant (**P=0.006). (**f**) Representative images for the quantification in **e** (*t*_irr,_ timepoint of irradiation; *t*_120s_, 2 min after irradiation). Images are displayed in pseudocolors for better illustration. (**g**) H2B and H3 bind tighter to chromatin in absence of CDC73 at a salt concentration of 0.6 mm NaCl. GAPDH and Ku70/86 are shown as soluble controls. Ratios for CDC73 knockdown and H2B and H3 in the soluble fraction are quantified and summarized in the table.

Recently the ubiquitin ligases RNF20/40 were implicated in DNA DSB repair by monoubiquitinating H2BK120 [8, 9]. Similar to our observations on the loss of CDC73, loss of RNF20 and RNF40 has been shown to lead to chromosomal instability [23]. Furthermore, CDC73 has been shown to directly bind to RNF20/40 [7]. We reasoned that loss of CDC73 might lead to a defect in H2BK120ub and therefore limit the accessibility of repair factors to damaged chromatin (interfere with efficient 5′ end resection). Interestingly, we found that CDC73 binds to H2B and H3 in co-immunoprecipitation (co-IP) experiments (Figure 5a), which we confirmed by IP–mass spectroscopy (IP–MS) (Supplementary Table S6). Furthermore CDC73 silencing lead to a decrease in monoubiquitinated H2B in cells subjected to replication stress with HU (2 mm) (Figure 5b). This finding correlated with decreased levels of H2BK120Ub also in unstressed cells, which was rescued by re-expression of a siRNA-resistant CDC73 (Figure 5c).

These results indicate that CDC73 may have a role in H2B-mediated chromatin remodeling, and potentially H2B release at sites of DSBs. To study H2B release from chromatin directly after DNA damage induction we performed laser irradiation experiments in H2B-GFP expressing U2OS cells [24]. H2B-GFP dynamics were recorded in live-cells immediately after irradiation. Induction of DNA damage was determined by red fluorescent protein–XRCC1 recruitment (Figure 5d). Strikingly, CDC73 silencing significantly reduced the migration speed of H2B away from the site of damage immediately after irradiation. Expression of a H2BK120 mutant protein resulted in the same decrease promoting our hypothesis (Figure 5e and f). To directly test the release of H2B from the nucleosome, we did a nucleosome stability assay [25]. At a salt concentration of 0.6M NaCl, we eluted H2B and H3 from the chromatin in control cells. In contrast, we recovered less soluble H2B and H3 in cells depleted for CDC73 (Figure 5g). We conclude that on loss of CDC73, monoubiquitination of H2B and subsequent efficient eviction of H2B from the chromatin is impaired.

### CDC73s role in DSB repair is independent of transcriptional elongation

**Figure 6:**
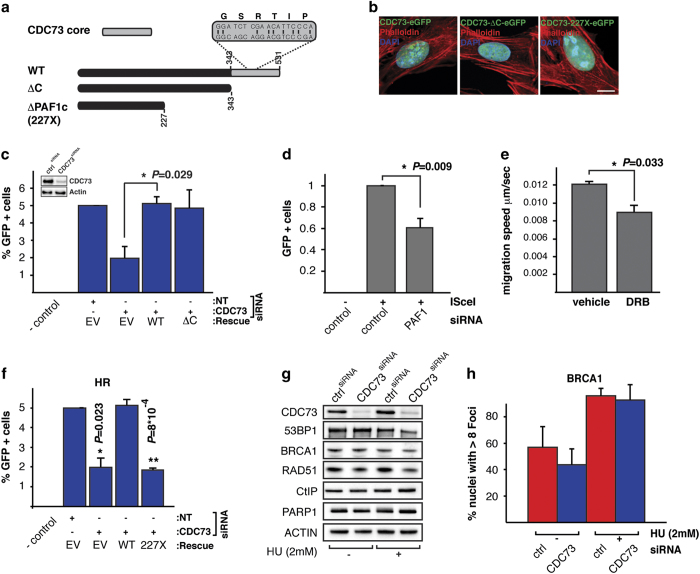
The role of CDC73 in HRR is independent of its transcriptional function. (**a**) Illustration of the siRNA-resistant CDC73, the core mutant (ΔC) and the 227X mutant. (**b**) Immunofluorescence of the eGFP-tagged constructs in U2OS cells in combination with DAPI (blue) and Phalloidin (red). (**c**) The defect in HRR as measured by the DR–GFP assay can be rescued by re-expression of a WT (*P=0.029) and the ΔC mutant. (**d**) PAF1 silencing does reduce HRR (*P=0.009). (**e**) DRB treatment (50 μm, 2 h) has the same effect on H2B mobility as CDC73 silencing (*P=0.033). (**f**) The CDC73-227X mutant does not rescue HRR in the DR–GFP assay (**P=8×10^-4^). (**g**) CDC73 knockdown does not affect the expression of a number of essential genes for DNA repair and has no effect on the recruitment of BRCA1 (**h**). For all experiments (*n*⩾2). Average and s.d. are plotted. *P*-values are calculated with Student’s *t*-test and scale bars represent 50 and 10 μm.

**Figure 7:**
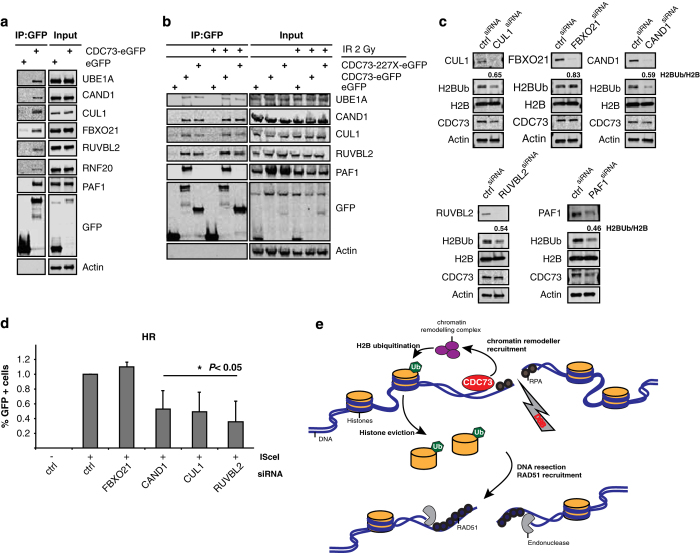
CDC73 interacts with a number of chromatin-remodeling factors. (**a**) Co-IP with CDC73 confirmed its interaction with UBE1A, CAND1, CUL1, FBXO21, KU86, RUVBL2 and RNF20. PAF1 serves as a positive control and actin as a negative control. (**b**) The interactions are unchanged on IR treatment (2 Gy). (**c**) siRNA-mediated silencing of FBXO21, RUVBL2, CAND1, CUL1 and PAF1 leads to a decrease in H2BK120Ub. Ratios H2BK120Ub:H2B are indicated. (**d**) siRNA knockdown of CAND1, CUL1 and RUVBL2 leads to a similar decrease in homologous recombination repair as CDC73 knockdown (s.d. of average; more than three repeats). (**e**) Model describing the mode of action of CDC73 at DSB. CDC73 recruits chromatin-remodeling components that mediate H2BK120Ub and subsequent eviction of H2B from chromatin. The decondensed state of the chromatin allows repair enzymes to engage with DNA and proceed with resection and downstream DNA repair.

To functionally determine essential domains in CDC73 required to mediate DNA repair, we generated siRNA-resistant constructs of CDC73 harboring deletions in either the CDC73 core domain (ΔC), which is required for transcriptional elongation but does not affect PAF1c assembly [26], or the 227X mutation, a mutation found in patients which codes for a protein unable to bind PAF1c [5] (Figure 6a). All constructs are expressed and in the nucleus (Figure 6b). To study the requirement of these domains specifically for HRR, we depleted endogenous CDC73 and reconstituted cells with the rescue constructs. HRR efficiency was determined by the DR–GFP assay [27]. Not only did the WT construct completely rescue the defect in HRR but also the ΔC mutant fully reconstituted HRR activity (Figure 6c). These findings indicate that the effect of CDC73 depletion on DSB repair is specific and that the transcriptional function of CDC73 in immediate DNA repair is not important. Supporting this finding, we did not find other PAF1c components Leo1, Rtf1 or Ctr9 in our screen for RAD51 foci (Supplementary Figure S1E). However PAF1 knockdown does affect recombination efficiency as determined in the DR–GFP reporter assay (Figure 6d) and affects RAD51 recruitment in our screen (Supplementary Figure S1E). Supporting a necessity of CDC73 in the PAF1c or binding to RNA pol II for the repair of H2BK120 does short time (2 h) treatment of cells with the transcription inhibitor DRB, which blocks RNA pol II phosphorylation, lead to the same effect as CDC73 silencing in the H2B eviction experiment (Figure 6e). This finding is furthermore supported by the fact that the CDC73-227X mutant completely fails to rescue the loss of endogenous CDC73 in the DR–GFP assay (Figure 6f). Loss of CDC73 in our experiments did not affect the abundance of essential repair proteins like 53BP1, CtIP, PARP1, BRCA1 and RAD51, supporting our observation that the repair defect caused by loss of CDC73 is not due to blocked transcription of essential repair genes (Figure 6g and h). In search for proteins that could account for the HRR defect in the absence of CDC73, we performed Immunoprecipitation of eGFP-tagged CDC73 followed by mass spectrometry. As a measure for the quality of our experiment, we detected all PAF1c components to bind to CDC73-eGFP but not to eGFP alone. Interestingly, we did find a number of chromatin-remodeling factors belonging to the SCF/Cullin and INO80 complexes. To confirm the hits from the mass spectrometry experiments, we repeated the Immunoprecipitation followed by western blot. Besides the confirmation of the interaction of CDC73 with RNF20, we confirmed interaction with UBA1, RUVBL2, CUL1, CAND1 and FBXO21. RUVBL2 is a component of the INO80 and NuA4 chromatin-remodeling complex, and CUL1, CAND1, and FBXO21 are SCF complex components (Figure 7a). The interactions were not altered after DNA damage (IR, 2 Gy), indicating that these interactions are also involved in canonical transcription (Figure 7b). The 227X mutant that retains only the 227 most N-terminal aminoacids still interacts with the candidate proteins, demonstrating that the PAF1c interaction domain and the chromatin-remodeling domain are separated (Figure 7b). A direct role for FBXO21, CUL1, CAND1 and RUVBL2 in H2B ubiquitination has not been described yet. We therefore checked the level of H2BK120Ub by western blot and detected a decrease on siRNA-mediated silencing similar to the decrease after CDC73 siRNA silencing (Figure 7c), which was also reflected in a decrease in the ability to repair DSBs in the HRR reporter cell line (Figure 7d). The effects are very similar to the decrease in H2BK120Ub and HRR activity after CDC73 silencing, and also PAF1 silencing affects H2BK120Ub to a similar extent. The effect on the stability of CDC73 itself is, however, only minor after silencing of CUL1, CAND1 and PAF1 (Figure 7c).

Summarizing our results, we propose a model in which CDC73, as a component of the PAF1c recruits a number of chromatin-remodeling factors which mediate H2BK120Ub to ensure efficient release of H2B from the chromatin and allow DNA repair factors to access the DNA for the repair of DSBs. This effect is dependent on CDC73 engaging with the PAF1c but is independent of transcription. This mechanism serves as a rapid sensor of DNA damage at transcribed regions and is essential for faithful repair by HR.

## Discussion

Here we present a genome-wide siRNA screen for regulators of HRR-mediated DNA repair activated after IR. We identified new factors involved in HRR and present the results of our screen as a resource to the scientific community.

In general, HRR activated after IR- and CPT-induced lesions employ similar proteins for the subsequent formation of RAD51 foci, and proteins involved in HRR triggered by restriction endonucleases are similar to those induced by IR. So it appears as no matter how HRR is initiated the subsequent repair is overall similar. Also, IR-induced RAD51 foci form relatively late in S-phase, at a time when a majority of DNA has been replicated (Figure 1d). This is likely a reflection of HRR being triggered by secondary DSBs after IR, occurring when replication forks collide with complex (clustered) lesions [15].

We present the characterization of the tumor suppressor CDC73 as an important novel regulator of HRR-mediated DNA repair. Loss of CDC73 leads to increased genomic instability which is likely to be due to paused RNA polymerase, increasing the likelihood of collisions between RNA polymerase and the replication fork. This adds CDC73 to the long list of DSB repair tumor suppressor genes preventing cancer by promoting genome stability.

There is multiple emerging evidence that processes in transcription are closely linked to DNA repair. It is conceivable that decondensation of chromatin and accessibility of the DNA are equally important for both processes. For example, inhibition of proteasomal activity was shown to affect homologous recombination [28], and loss of RNF20 results in genomic instability [23]. Also monoubiquitination on Histone 2B was directly linked to chromosomal stability[29] and it was recently shown that the ubiquitin ligases RNF20 and RNF40 are required for the repair of DSBs [8, 9].

We describe here a novel function for CDC73 in promoting and directing Histone 2B monoubiquitination for the efficient eviction of H2B and DSB repair that is independent of CDC73s transcriptional function. Loss of CDC73 results in decreased levels of H2BK120Ub during DNA repair, less soluble H2B and H3 and a reduced mobility of H2B directly at sites of irradiation (Figure 7e). We discover the interaction of CDC73 with members of the SCF/CUL1 chromatin-remodeling complex and the chromatin remodeler RUVBL2 that is mediated by the N-terminal domain of CDC73. The SCF complex as well as RUVBL2 have been previously implicated in the repair of DNA DSB with RUVBL2 being a direct target of ATM and ATR [30–36]. Initial recruitment of repair factors is not impaired in the absence of CDC73 as neither expression of genes required for resection is affected nor the recruitment of the single-stranded DNA-binding protein RPA and upstream factors. We do, however, observe a defect in the clearance of RPA from single-stranded DNA, a reduction in RPA S33 phosphorylation and subsequently impaired recruitment of RAD51 and repair of DSBs. These results lead us to conclude that on loss of CDC73, the nucleosomes remain in a condensed state presenting a physical barrier for the repair machinery. The finding that the phosphorylation of H2AX and the subsequent recruitment of 53BP1 and RPA to sites of DSBs are not impaired but the resolution of those foci is strongly delayed furthermore supports this statement. We also conclude that the RNF8/RNF168 branch of the DNA damage response[1] is unaffected by loss of CDC73. Instead, the repair machinery is unable to proceed when it encounters the tightly packed chromatin and fails to conclude the repair process (Figure 7e). Another possible scenario could be RUVBL2-mediated nucleosome sliding and CDC73/PAF1c–RNA pol II backtracking to expose damaged DNA as it was recently described for UvrD in *Escherichia coli* [37].

Taking together, here we present a novel role of CDC73 in DNA repair and genome stability, which is independent of its transcriptional function. We identified a number of chromatin-remodeling factors to interact with CDC73 and that their function on chromatin in this context depends on the recruitment to the PAF1c and thereby to transcribed DNA. We propose that this newly identified function of CDC73 in Histone eviction at sites of DNA damage together with the previously shown role of CDC73 in regulating oncogene expression is important to prevent tumorigenesis.

## Materials and Methods

### Cell culture

U2OS cells (ATCC, United Kingdom (U.K.), Guernsey, Ireland, Jersey and Liechtenstein #HTB-96) were cultured in DMEM with 10% fetal calf serum, penicillin (60–100 μg ml^−1^), streptomycin (100 μg ml^−1^) at 37 °C containing 5% CO_2_, in a humidified incubator. Cells were regularly checked for mycoplasma contamination (MycoAlert, Lonza Walkersville, Walkersville, MD, USA).

### Antibodies

The following antibodies were used: ATR (sc1887 N19); RAD51 (sc8349); RPA (Cell Signaling Technology, Danvers, MA, USA, 4E4); P-ATM (sc47739); H2B (ab52484); H2BK120Ub (Cell Signaling Technology, D11); H3 (sc10809); Actin (ab6276); CDC73 (Cell Signaling Technology, D38E12); P-RPA (Bethyl Laboratories NB100-S44); P-ATR (Cell Signaling Technology, S428); P-Chk1 (Cell Signaling Technology, 133D3); Chk1 (Cell Signaling Technology, 261DS); 53BP1 (ab36823); GFP (sc8334); PARP1 (sc7007 F2); KU70 (sc17789 E5); CtIP (Bethyl Laboratories, Inc., Montgomery, TX, USA,A300-488A); BRCA1 (sc642); γH2AX (Millipore, Merck Chemicals and Life Science AB, Solna, Sweden, JBW301); PAF1 (ab137519); GAPDH (sc365062); KU86 (sc528); Fibrillarin (ab5821); UBA1 (Cell Signaling, 4890); CAND1 (Bethyl Laboratories, Inc. A302-901A-T); RNF20 (Cell Signaling Technology, 9425); RNF40 (Bethyl Laboratories, A300-719A-T); RUVBL2 (Bethyl Laboratories, A302-536A-T); CUL1 (Bethyl Laboratories, A303-373A-T); FBXO21 (ab179818); Flour-555 (A21434, Invitrogen, Life Technologies Europe BV, Stockholm, Sweden); Alexa Flour-488; Phalloidin 594 (Sigma-Aldrich Company Ltd, Dorset, UK, 51927); mouse, rabbit, rat horseradish peroxidase-conjugated ab (Abcam, Cambridge, UK); mouse and rabbit IRDye-conjugated ab (Licor, Lincoln, NE, USA).

### Generation of the U2OS-DR–GFP and non-homologous end-joining cell lines

The U2OS-DR–GFP cell line was generated by transfecting 1×10^6^ U2OS cells with 4 μg of the DR–GFP vector. Stable cell lines were selected with 3 μg ml^−1^ puromycin and single clones were picked, expanded and later checked for GFP expression with or without transfection with an I-SceI endonuclease-expressing vector. A cell line with low (<0.1%) spontaneous expression and high number of GFP-positive cells (>3%) after I-SceI-induction was selected. The same protocol was followed for generation of the non-homologous end-joining expressing cell line (Addgene 40026).

### FACS analysis

DR–GFP: 20 000 Cells were seeded in 12 well plates. 24 h later cells were transfected with a ISceIendonuclease plasmid (150 ng, JetPEI (Polyplus, Bioparc, Illkirch, France)) to induce DSBs. GFP-positive cells were analyzed using FACS (Beckman Coulter Navios, Archimedesvägen, Bromma, Sweden) 48 h after I-SceI transfection as described previously [2, 27]. For rescue experiments, cells were first transfected with 5 nm siRNA 24 h after seeding. After 8 h, DNA was transfected: CDC73 in combination with I-SceI (150 ng each). Cells were collected for FACS 48 h later. For cell cycle analysis, cells were labeled with BrdU (20′) (BD Pharmingen, BD Biosciences, San Jose, CA, USA, BrdU Flowkit) and counterstained with propidium iodine. Data were analyzed with Kaluza software (Beckman Coulter AB, Bromma, Sweden). More than 20 000 cells were analyzed per experiment.

### siRNA screen

The siRNA screen using the Dharmacon (VWR International - Sweden AB, Stockholm, Sweden) human siGENOME siRNA library (druggable, drug targets and genome subsets) was performed using solid-phase transfection plates [5, 38, 39] from CytoPathfinder, Tokyo, Japan. These plates contained siRNA (siGENOME SMARTpool, four individual siRNA sequences per gene, Thermo Scientific), transfection reagent (DharmaFECT 1, #T-2001-02 Thermo Fisher Scientific, Waltham, MA, USA) and accelerator (CytoPathfinder) in every well of 384-well plates, except outer columns. U20S cells (1 000 cells per well) were reverse transfected onto 384-well plates at a final siRNA concentration of 10 nm and incubated for 68 h when plates were irradiated with 4 Gy (1.96 Gy min^−1^, Cs^137^). At 72 h after siRNA transfection, cells were fixed and subsequently stained with RAD51 antibody and nuclear stain (DAPI). All liquid handling steps were performed on a Janus Automated Workstation (Perkin-Elmer Waltham, MA, USA) and automated imaging (four images per channel, two channels per well)) was conducted using an IN Cell Analyzer 1000 (GE Healthcare, Danderyd, Sweden). Images analysis was done using the IN Cell Investigator software (GE Healthcare Life Sciences, Uppasala, Sweden) to score RAD51 foci count as well as DAPI intensity per cell. The genome-wide siRNA screen was run in technical and biological duplicates.

For the rescreen of the 87 selected siRNAs, Ambion siRNA (pool of three individual siRNA sequences per gene) was used from the . For all 3 validation assays, U2OS cells (6000 cells per well on 96-well plate) or U2OS-DR–GFP cells (10 000 cells per well) were reverse transfected with siRNA at a concentration of 10 nm. DharmaFect 1 was used as a transfection reagent at a 1:1 000 dilution. Control siRNAs were the same for both primary screen and validation assays, negative control—non-targeting siRNA from Qiagen, Valencia, CA, USA (#1022076)—and positive control—RAD51 siRNA (Thermo Scientific #M-003530-04 and Ambion, Life Technologies Europe BV, Stockholm, Sweden, #4392420). All assays were run in duplicate.

### 3D-*B* score normalization

We developed and applied a modification of the *B* score calculation as described in Malo *et al.* [40, 41], that we termed 3D-*B* score normalization. We first estimate the systematic column, row and plate effects by performing a three-way median polish over the 3D matrix obtained when stacking the matrices obtained from the individual plates on top of each other. This allows for more robust determination of the systematic row and column effects are obtained due to the fact that they are estimated using the whole data set and not on a per-plate basis. Another advantage is that an integral estimation of the plate-to-plate variation. Systematic deviations were substracted from our data set to obtain a 3D matrix with residuals, that is, data that cannot be explained by the systematic effects estimated and therefore must represent true data and noise. Finally, the residuals are scaled to 1.4826 times the median absolute deviation of the median, which in a large sample of a normally distributed population is an estimator for the population s.d.

### Immunofluorescence

Screen: cells in either 384-well or 96-well plates were fixed using 4% paraformaldehyde in PBS-T0.1 (0.1% Triton X-100 in phosphate-buffered saline (PBS)) for 15 min. Cells were washed with PBS twice, then PBS-T0.3 (0.3% Triton X-100) before blocking in 3% bovine serum albumin (in PBS). Incubation with primary antibody overnight at 4 °C and with secondary antibody for 1 h at room temperature. All washes with PBS, 3×10 min. DNA was stained with DAPI (#D9542, Sigma) or To-Pro-3-Iodide (Invitrogen 642/661) and images were acquired using an IN Cell Analyzer 1000.

For confocal imaging, cells were grown on cover slips. After treatment, cells were washed in PBS, pre-permeabilised in ice cold PBS 0.5% Triton X-100 for 2 min and fixed in PBS 4% PFA. Immunostaining was done according to standard protocols and images were acquired on a Zeiss LSM 780 (Zeiss, Jena, Germany). For high content microscopy, cells were grown in 96-well plates (BD Falcon; Fisher Scientific, Pittsburgh, PA, USA) and images were acquired on an Operetta system (Perkin-Elmer). Images were analyzed with Columbus software (Perkin-Elmer).

For live imaging, cells were seeded on imaging plates (Fluoro dish, World Precision Instruments, Sarasota, FL, USA) and transfected with siRNA. After 48 h, cells were incubated in Hoechst (1:1 000) for 5 min and washed in phenol red-free medium. H2B–GFP experiment: Laser irradiation was done after 6 frames (stripe width 6 pixel, scan speed 12.65 μs per pixel, 20% laser power (405 nm laser), ×40 objective with ×6 digital zoom) in a heated (37 °C, 5% CO2) and humidified chamber on a Zeiss LSM 780 confocal laser scanning microscope equipped with a ultraviolet-transmitting Plan-Apochromat ×63/1.40 Oil DIC M27 objective (Zeiss). Images were acquired for 2 min. H2B plasmid was obtained from Addgene (Cambridge, MA, USA; 11680). Data were quantified using the plot profile function in ImageJ (University of Wisconsin-Madison, Madison, WI, USA). More than 10 cells per experiment were analyzed and the experiment was repeated at least 3 times.

### Gene enrichment and network analysis

Statistically overrepresented pathway annotations for the initial list of candidate genes (*n*=200) were determined using the hypergeometric probability distribution. *P*-values were adjusted for multiple testing using the strategy described by Benjamini and Hochberg [6, 42]. Pathway annotations were collected from the KEGG database (as of February 2010) [7, 43]. The obtained data set comprised 236 pathways. For further analysis, the candidate genes were mapped to the human FunCoup network (version 1.0) [8, 9, 16, 17] only including links with a confidence cut-off ⩾0.5. The network was clustered into local sub-networks using MGclus[10, 18] and functionally annotated as described above.

### Comet Assay

U2OS cells were seeded on six well plates (50 k cells per well). siRNA (5 nm) was transfected the day after with Interferin (Polyplus). Cells were collected after 48 h and washed with 1× PBS. Cells were re-suspended in 1× PBS at a concentration of ~5×10^5^ cells ml^−1^. About 50 μl cell suspension was mixed with 250 μl 1.2% low-melting agarose at 37 °C. The cell suspension was added to pre-warmed (37 °C) agarose-coated fully frosted slides (Thermo Fisher Scientific) and a coverslip was added on top. Slides were kept on ice for 10 min before removing the coverslip and incubated in lysis buffer (10 mm Tris pH 10.0, 2.5 m NaCl, 0.1 M EDTA, 10% DMSO and 1% Triton X-100) at 4 °C overnight in the dark. The next day, slides were incubated in alkaline electrophoresis buffer (0.3 N NaOH, 1 mm EDTA) for 30 min. Electrophoresis was run at 300 mA, 25 V for 30 min in electrophoresis buffer using a Comet Assay tank (Thistle Scientific, Glasgow, UK). Slides were washed in neutralization buffer (0.4 m Tris-HCl pH 7.5) and counterstained with 5 μM YOYO-1 dye (Invitrogen). Images were acquired with a ×20 or ×10 objective in a Zeiss LSM 510 confocal microscope and quantified using CometScore software (TriTek Corp., Sumerduck, VA, USA). At least 30 comets per sample were analyzed. Tail moment is calculated as percent DNA in the tail multiplied by the tail length.

### Molecular cloning

CDC73 ORF was amplified and cloned into pCDNA3.1. C-terminal eGFP tag was introduced by PCR. The CDC73 siRNA-resistant construct as well as the different mutations were cloned by fusion PCR. The CDC73ΔC construct carries a deletion of the C-terminal 188 aminoacids and the CDC73-227X mutant lacks the C-terminal 304 aminoacids. H2B–GFP was ordered from Addgene (11680) [24] and red fluorescent protein–XRCC1 was described earlier [12].

### Co-Immunoprecipitation

About 1.5×10^6^ HEK293T cells were seeded in 10 cm^2^ dishes. GFP-tagged CDC73 constructs were transfected the next day (6 μg per plate) according to manufacturer's protocol (JetPEI). After 48 h, cells were collected and lysed in buffer containing DNAseI (1 μg μl^−1^; 2.5 mm MgCl_2_). IP was carried out using the GFP trap kit (ChromoTec, Planegg-Martinsried, Germany). For IP–MS, the protein was run on a SDS–PAGE and the gel was stained with Coomassie brilliant blue (Bio-Rad). The protein mixture was in gel digested with Trypsin (Promega, Madison, WI, USA) and analyzed on an Orbitrap XL (Thermo Fisher Scientific). Peptides were annotated with MASCOT and peptides with a protein score <31 were discarded.

### Western blotting

Western blotting was carried out following standard protocols with Bio-Rad gels and the Trans-Blot Turbo transfer system (Bio-Rad). Cells were lysed in RIPA buffer 20 min on ice followed by sonication with a needle sonicator (Hielscher Ultrasonics GmbH, Teltow, Germany, UP100H; 70% amplitude; 0.7 cycle; 10 cycles). Images were taken at a ChemiDoc XRS system (Bio-Rad) or an LI-COR Odyssey FC (LI-COR, Lincoln, NE, USA). Quantification was done with ImageStudio (LI-COR).

### Cell transfections

Transfections were carried out according to manufacturer's protocols. For DNA transfection, jetPEI (Polyplus) was used, and for siRNA transfections interferin was used (Polyplus). siRNA sequences are control; CDC73#1 5′-GGATCTCGAACACCCATTA-3′; CDC73#2 5′-CTATCAAGACTGATCTAGA-3′; CDC73#3 5′-GACCAGTGTTCTTACGGTT-3′; CDC73#4 5′-TTCAGTGTCATACCATGGTAGGTTC-3′; CDC73#5 5′-GGGCACTGCAATTAGTGTTACAGTA-3′, PAF1#1 5′-ATCACCTGAGCACATGGATTGATCC-3′, PAF1#2 5′-TAATCATGGCCTGAGACATCATCTC-3′. CDC73 siRNA #4 and #5 and PAF1 siRNAs were previously described in refs [13 , 44]. RAD51 siGENOME SMARTpool (Thermo Scientific M-003530-04); RAD51 Silencer Select (Ambion, Invitrogen 4392420); FBXO21 (ON-TARGET plus (23014) SMARTpool, Thermo Scientific); FBXO22 (ON-TARGET plus (26263) SMARTpool); CAND1 (ON-TARGET plus (55832) SMARTpool); CUL1 (ON-TARGET plus (8454) SMARTpool); RUVBL2 (ON-TARGET plus (10856) SMARTpool).

## Notes

Supplementary Table S1

Supplementary Table S2A

Supplementary Table S2B

Supplementary Table S3A

Supplementary Table S3B
