# Supplementary figures and images for: A genome-wide IR-induced RAD51 foci RNAi screen identifies CDC73 involved in chromatin remodeling for DNA repair
Source: Cell Discov. 2015 Dec 1;1:15034–. doi: 10.1038/celldisc.2015.34 (PMC4860774; doi:10.1038/celldisc.2015.34)

Figure S1

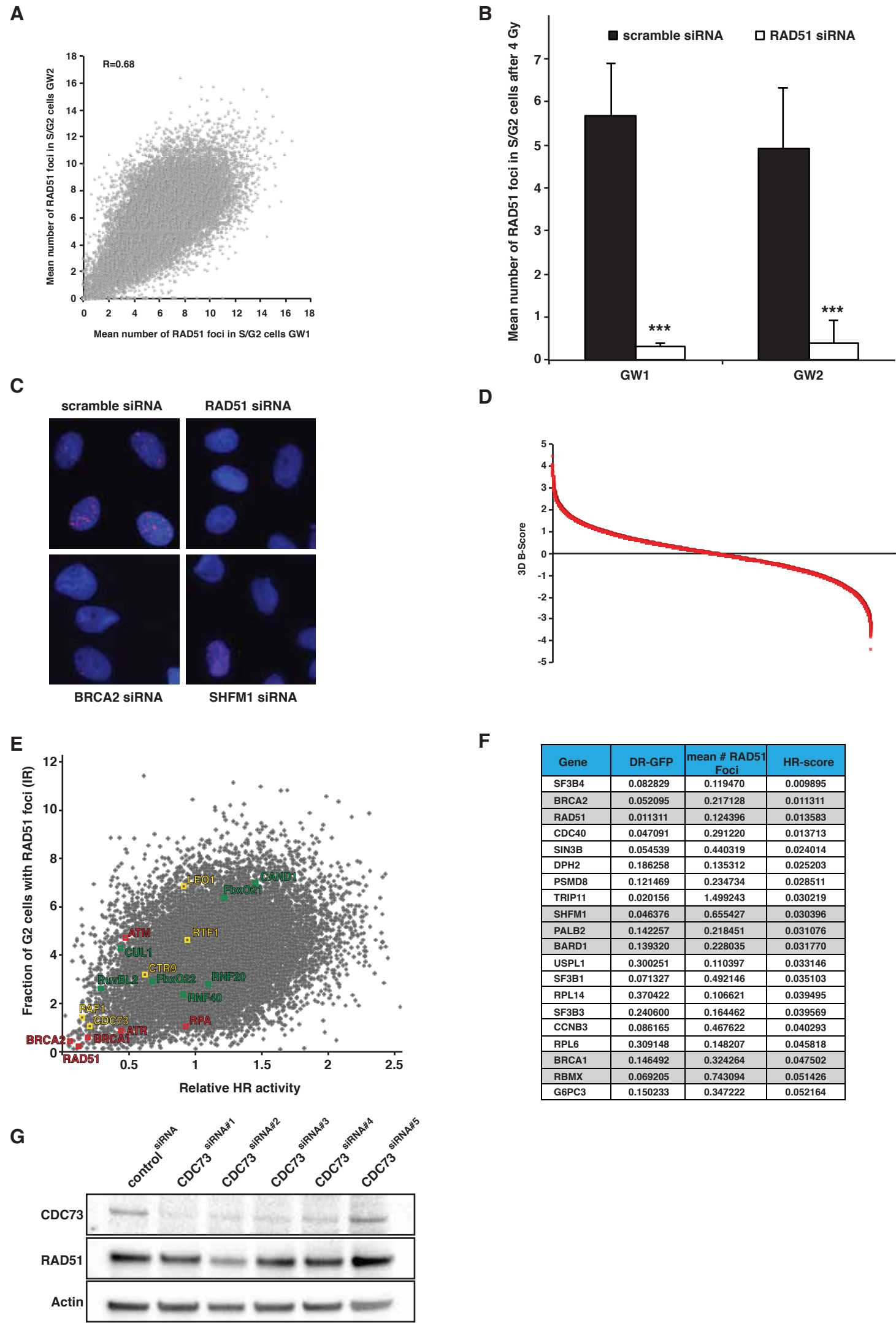

Supplement: Supplementary Figure S1 [file celldisc201534-s9.pdf]

Figure S2

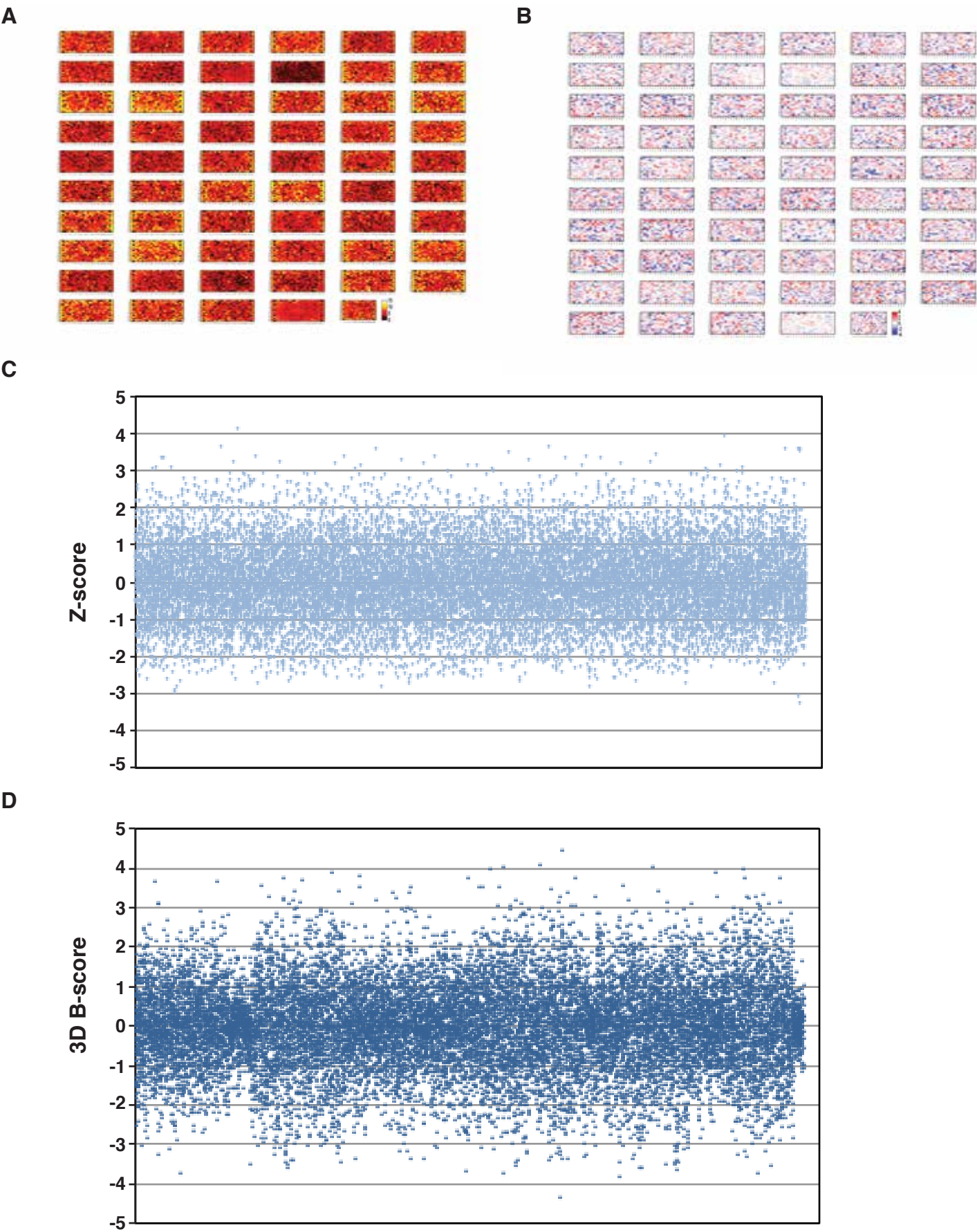

Supplement: Supplementary Figure S2 [file celldisc201534-s10.pdf]

Figure S3

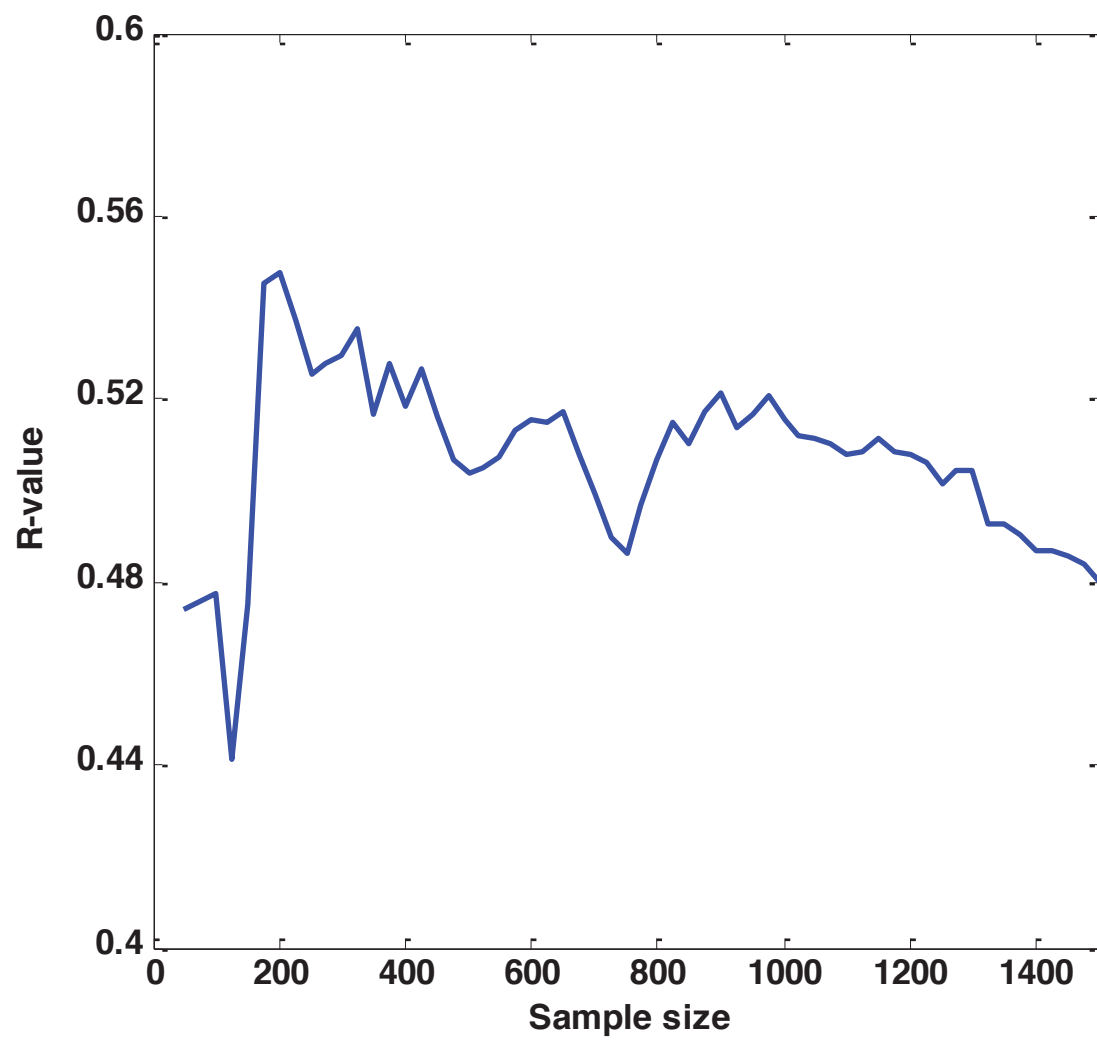

Supplement: Supplementary Figure S3 [file celldisc201534-s11.pdf]

Figure S4

A

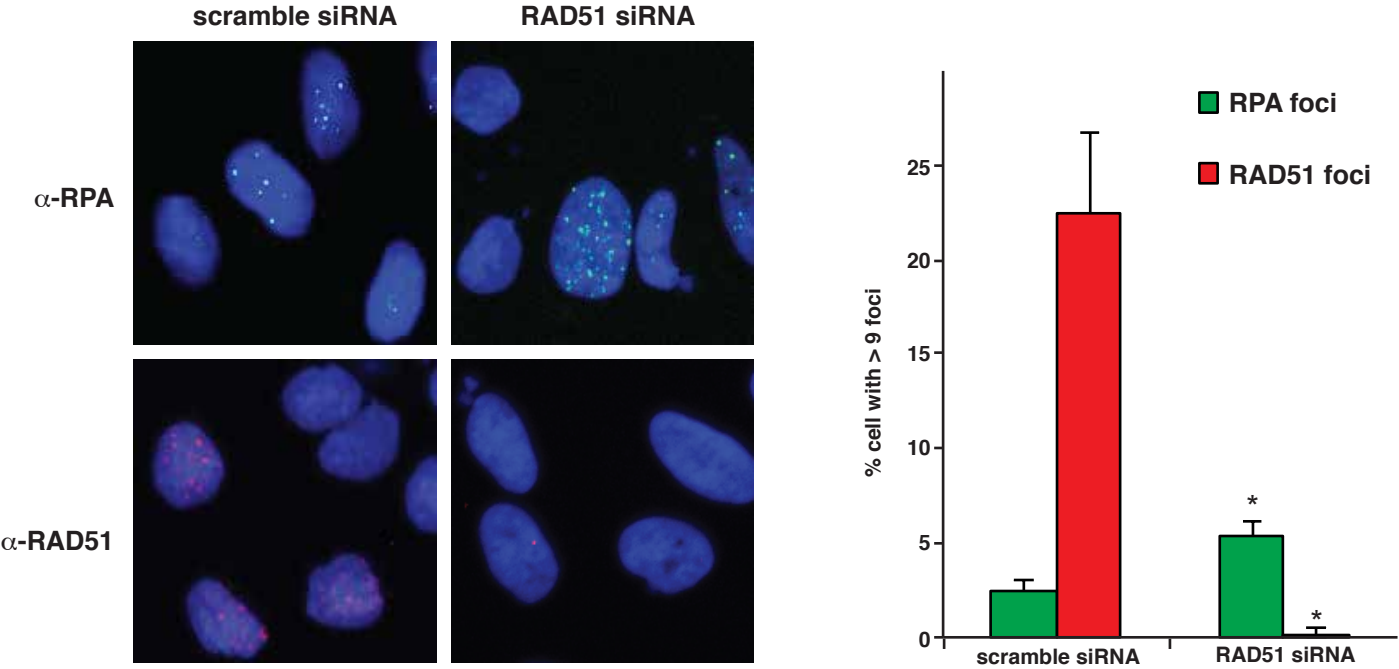

B

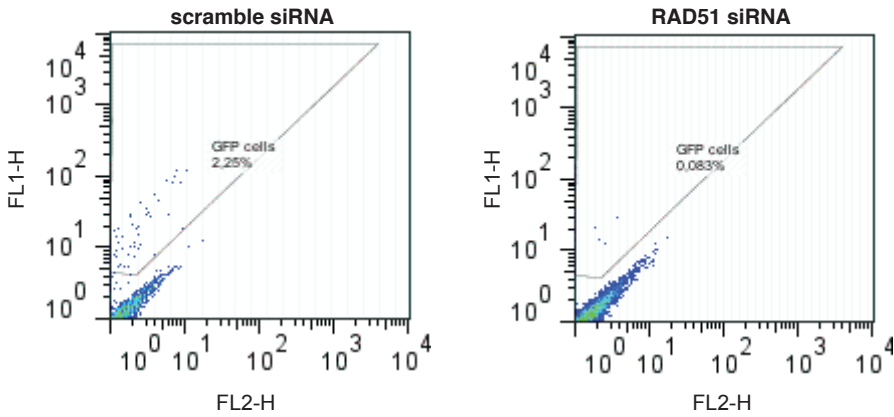

Supplement: Supplementary Figure S4 [file celldisc201534-s12.pdf]
