# Supplementary material for: A genome-wide IR-induced RAD51 foci RNAi screen identifies CDC73 involved in chromatin remodeling for DNA repair
Source: Cell Discov. 2015 Dec 1;1:15034–. doi: 10.1038/celldisc.2015.34 (PMC4860774; doi:10.1038/celldisc.2015.34)
Supplement: Supplementary Information [file celldisc201534-s13.doc]

**Supplementary figures.**

Supplementary Figure S1. (A) Correlation of mean RAD51 foci values from the duplicate runs, GW1 and GW2 (GW: genome-wide). The correlation coefficient is R=0.68. (B) Variation in RAD51 foci levels for control siRNAs in the whole plates set. Bars and error bars show mean RAD51 foci levels and standard deviation for negative (SCRAMBLED) and positive (RAD51) control siRNAs for 58 plates in each duplicate run. (***) denotes a statistically difference of p<0.001 in Student’s t-test between negative and positive control for each screen run. (C) Representative merged images of RAD51 foci in cells treated with different siRNAs. DAPI is shown in blue and RAD51 foci in red. Labels show the different siRNA treatments. (D) 3D-B score of all siRNAs from the first screen run (GW1). siRNAs with low levels of RAD51 foci have a negative 3D-Bscore. (E) Correlation of our screen with the DR-GFP screen from Adamson et al., 2012 [4]. Correlation coefficient is 0.35. A small selection of known HR genes is indicated in red, Ubiquitin ligases RNF20/40 in green and PAF1c components in yellow. (F) Table listing the proteins that suppress both HR and RAD51 foci formation most effectively. HR-score is calculated as the product of the scores in the individual screens. (G) Western blot for CDC73 and RAD51 after silencing of CDC73 with 5 different siRNA sequences.

Supplementary Figure S2. Normalization using 3D-B score. (A) Heat map showing mean number of RAD51 foci in S/G2 cells for all 58 plates in the siRNA library. Mean values ranging from 0 to15 is displayed from dark red to light yellow, respectively. (B) Data was normalized using a 3D-B score normalization on the mean number of RAD51 foci in S/G2 cells for the whole data set. Colour range of 3D-B score (blue to white to red) goes from -4 to 4. (C) Dot plot of the z-score for the genome-wide siRNA screen. (D) Dot plot of the normalized 3D-B score values for the whole data set resulting in (A).

Supplementary Figure S3. Determining sample size of hit list. Correlation coefficient was calculated by plotting mean RAD51 foci in SG2 cells for GW1 vs. GW2, sorted based on merged rank number of 3DB-score. Sample size started at n=50 and increased by 25 for each calculation. Best correlation (R=0.5477) gave a sample size of n=200.

Supplementary Figure S4. Results of positive and negative control siRNAs in the three validation assays. (A) RPA and RAD51 foci formation after irradiation (4Gy) in SCRAMBLED and RAD51 siRNA-treated cells. RPA foci (upper panel) are show in green, RAD51 foci (lower panel) in red and DAPI in blue. Bar graph shows mean foci values for SCRAMBLED- and RAD51-siRNA treated cells. One-star (*) denotes a statistical significant difference of p<0.05 in Student’s t-test compared to SCRAMBLED siRNA-treated cells. (B) Dot plot of GFP-positive cells after I-SceI-induced DSBs in cells treated with SCRAMBLED or RAD51 siRNA.

**Supplementary tables.**

Supplementary Table S1. Genome-wide screen data. All data calculated from analysis of images for respective screen run (GW1 or GW2). For each siRNA pool, information about targeted gene (Gene Symbol), Gene ID, Vendor ID and GeneBank Accession number is given. For each screen run, results from image analysis are shown, i.e. toxicity (relative siRNA control –treated cells), total number of cells counted, %cells in S/G2 phase, mean number of RAD51 foci in S/G2 cells, % S/G2 cells with >9 RAD51 foci and 3D-B score value. Last column show Merged rank number from 3D-B score list of each duplicate screen run.

Supplementary Table S2A. Result of the cluster analysis. For each cluster, significant enriched KEGG-pathways, identified genes and the respective KEGG-annotation are shown.

Supplementary Table S2B. Result of the gene enrichment analysis showing pathways significantly associated with our 200 candidate genes.

Supplementary Table S3A. Filtering to reduce false-positives. Overlap of our candidate lists with hit lists from previous HR screens before and after network analysis.

Supplementary Table S3B. Result of statistical calculation (t-test) on the 87 rescreened siRNAs. Table shows both score (statistically significance, p< 0.05 or not) and actual p-values.

Supplementary Table S4. Data for the 87 siRNAs picked for rescreening using Ambion Silencer Select siRNA pools. For each siRNA pool, information about targeted gene (Gene Symbol), Gene ID, Vendor ID, siRNA sequences and GeneBank Accession number is given. Mean values and standard deviation from duplicate runs of each validation assay (RPA, RAD51 foci and DR-GFP assay) are presented. RAD51 and RPA foci values are shown as % cells with more than 9 foci, HR-activity in the DR-GFP assay is calculated as %GFP-positive cells of whole population.

Supplementary Table S5. Data for the 17 siRNAs that showed HR-defect in all 3 validation assays. Mean and standard deviations calculated from two independent experiments.

Supplementary Table S6. IP-MS results. CDC73-eGFP or eGFP was used for CoIP in HEK293T cells. Interacting proteins were separated on a SDS gel and subsequently processed for MS. Provided is a list of potential CDC73 interacting proteins.
